# The Effect of Cadmium on Sleep Parameters Assessed in Polysomnographic Studies: A Case–Control Study

**DOI:** 10.3390/jcm12123899

**Published:** 2023-06-07

**Authors:** Weronika Frosztega, Mieszko Wieckiewicz, Pawel Gac, Gabriella Lachowicz, Rafal Poreba, Grzegorz Mazur, Helena Martynowicz

**Affiliations:** 1 Department of Internal Medicine, Occupational Diseases, Hypertension and Clinical Oncology, Wroclaw Medical University, 50-556 Wroclaw, Poland; weronika.frosztega@gmail.com (W.F.); gabriella.lachowicz@umw.edu.pl (G.L.); rafal.poreba@umw.edu.pl (R.P.); grzegorz.mazur@umw.edu.pl (G.M.); helena.martynowicz@umw.edu.pl (H.M.); 2 Student Research Club No K133, Faculty of Medicine, Wroclaw Medical University, 50-367 Wroclaw, Poland; 3 Department of Experimental Dentistry, Wroclaw Medical University, 50-425 Wroclaw, Poland; 4 Division of Environmental Health and Occupational Medicine, Department of Population Health, Wroclaw Medical University, 50-345 Wroclaw, Poland; pawel.gac@umw.edu.pl

**Keywords:** cadmium, sleep architecture, polysomnography, REM sleep stage, obstructive sleep apnea (OSA), sleep bruxism (SB)

## Abstract

Cadmium is a heavy metal that accumulates in the body due to environmental and occupational exposure. The main form of environmental exposure to cadmium is related to cigarette smoking. The primary aim of this study was to evaluate the effect of cadmium on numerous sleep parameters with the use of polysomnography. The secondary aim of this study was to investigate if environmental exposure to cadmium is a risk factor for the intensity of sleep bruxism (SB). Methods: A total of 44 adults underwent a full night of polysomnographic examination. The polysomnograms were assessed according to guidelines set out by the American Academy of Sleep Medicine (AASM). The concentration of cadmium in the blood and urine was determined spectrophotometrically. Results: The polysomnographic examination confirmed that cadmium, age, male gender and smoking status are independent risk factors for an increase in the apnea–hypopnea index (AHI). Cadmium alters sleep architecture by favoring sleep fragmentation and decreasing the duration of the rapid eye movement (REM) phase of sleep. However, cadmium exposure is not a risk factor for the development of sleep bruxism. Conclusions: In summary, this study demonstrates that cadmium affects sleep architecture and is a risk factor for the development of obstructive sleep apnea; however, it does not affect sleep bruxism.

## 1. Introduction

Cadmium is a widespread heavy metal that is present naturally in the Earth’s crust [1]. Its biological half-life within the body, after environmental exposure, is estimated to be about 20 years [2]. In the last century, the industrial sector has most frequently used cadmium in the coating of iron and steel to prevent corrosion. Nowadays, this sector utilizes cadmium in the manufacture of batteries, pigments, coatings, plating substances and stabilizers for plastic. However, beyond the manufacturing sector, the most common form of exposure to cadmium is by means of inhalation and ingestion. Occupational exposure to cadmium relates to processes involving the heating of cadmium-containing materials [1].

Tobacco plants are known for absorbing heavy metals from the soil through their roots, accumulating them within their leaves which are ultimately used in the production of cigarettes. The single major significant source of cadmium exposure for the general population is tobacco smoking [3,4]. It has been established that smokers tend to have elevated cadmium concentrations in the blood, compared to their non-smoking counterparts [5,6]. It is worth noting, though, that non-smokers are also exposed to high quantities of cadmium that is present in water and food. This is particularly true of populations consuming diets that are high in rice and wheat content [5].

The kidneys are the major site of cadmium accumulation in the body. This in turn leads to nephrotoxicity and renal tubular damage, causing numerous negative health outcomes [3,7]. Cadmium has also been found to be neurotoxic. The recent literature states that cadmium has a strictly dose-dependent effect on neurons: Cadmium can gradually cause cell injury, cell death and organ failure at high doses. On the other hand, it can modulate specific mechanisms at low doses without significantly harming cells [8]. Neurodegenerative disorders, such as Alzheimer’s disease, Parkinson’s disease, amyotrophic lateral sclerosis, multiple sclerosis and myalgic encephalomyelitis, have previously been linked with cadmium-dependent neurotoxicity [9].

The symptoms of poisoning depend on the level of cadmium in the blood and may result in acute and chronic intoxication. It has been determined that cadmium is a type I carcinogen and acts as a proinflammatory cytokine inducer, leading to a chronic inflammatory response [3]. Cadmium exposure has been linked to the development of numerous illnesses, including osteoporosis [10], depressive symptoms [11,12], smoking-induced cardiovascular diseases [13] and pulmonary diseases [3]. Recently, cadmium exposure from tobacco smoking has been linked with type 2 diabetes mellitus [14]. Direct prolonged exposure to cadmium is known to be a major etiological factor of itai-itai disease (otherwise known as “ouch-ouch” disease) in Japanese female residents living near cadmium-contaminated rivers. Severe spine and joint pain, softening of the bones and kidney failure were the main findings in these patients. Several studies were carried out after reports of itai-itai disease came to light, in order to determine the levels of cadmium exposure that are a health threat [7,15]. Due to its adverse effects on human health, cadmium usage has been restricted worldwide under numerous regulations [16].

Worldwide culture-associated smoking habits have brought the attention of the scientific community to the substances present in cigarettes, ultimately raising environmental and health concerns. No studies have been published investigating the possible influence of cadmium on sleep architecture (rapid eye movement (REM), non-rapid eye movement (nREM)), respiratory function (apnea–hypopnea index (AHI) and saturation parameters) and movement disorders (bruxism episode index (BEI)). Due to the lack of research in this regard, the primary aim of this study was to evaluate the influence of cadmium on sleep architecture, by utilizing polysomnographic examination. The secondary aim of this study was to establish if exposure to cadmium increases the likelihood of developing obstructive sleep apnea (OSA) and if cadmium itself is a risk factor for sleep bruxism (SB).

## 2. Materials and Methods

### 2.1. Participants

This was a prospective, observational study of a total of 44 adults. The patients were admitted to the Department of Internal Medicine and Occupational Diseases, Hypertension and Clinical Oncology at Wroclaw Medical University, Poland, in order to undergo polysomnographic examination. The patients were referred to the sleep laboratory for examination, due to a suspicion that they may have a sleep disorder. These 44 subjects were admitted to the department between December 2020 and May 2021. Inclusion criteria were as follows: age over 18 years, written informed consent to polysomnographic examination and urine/blood sample collection. Patients were excluded from the study if they had neurological disorders and/or neuropathic pain, active inflammation, confirmed active malignancy, present or past occupational exposure to cadmium, severe respiratory and cardiac insufficiency, treatments affecting muscle function and sleep structure, severe mental disorders, cognitive disability and a lack of compliance during the study. All study participants declared no occupational cadmium exposure throughout their lifetime. The study was approved by the Ethics Committee at Wroclaw Medical University (approval no. KB-790/2022). Voluntary written informed consent was obtained from all patients prior to commencing the study. This study made use of the Strengthening the Reporting of Observational Studies in Epidemiology (STROBE) checklist [17].

### 2.2. Polysomnography

All subjects (*n* = 44) underwent polysomnographic investigation with a NoxA1 (NOX Medical, Reykjavík, Iceland) device. The recording was conducted between 22:00 and 6:00, in accordance with the patient’s natural circadian rhythm. Electromyographic electrodes were placed in line with guidelines set out by the American Academy of Sleep Medicine (AASM) [18]. Audio and video recordings together with electrocardiographic, electroencephalographic, electro-oculographic and electromyographic recordings were included, along with body position detection and thoracic and abdominal breathing activity. To measure saturation (SpO_2_%), pulse and plethysmography data, a WristOx2 3150 pulse oximeter (Nonin Medical Inc., Plymouth, MN, USA) was used. The respiratory, sleep bruxism (SB) and sleep parameters were gathered. Sleep parameters were obtained and classified pursuant to standard AASM criteria: non-REM 1 sleep stage (N1), non-REM 2 sleep stage (N2), non-REM 3 sleep stage (N3), REM sleep stage, arousal index (ArI), sleep latency (SL), wake after sleep onset (WASO), sleep efficiency (SE), apnea–hypopnea index (AHI), oxygen desaturation index (ODI) and snoring. SB parameters were also assessed: bruxism episodes index (BEI), phasic bruxism, tonic bruxism and mixed bruxism. A constant burst episode sustained over 2 s was categorized as a tonic episode. An episode including three or more bursts lasting over 2 s was categorized as phasic, and an episode showing the characteristics of both tonic and phasic episodes was categorized as a mixed episode of SB. SB episodes were defined as the rhythmic movements of the masseter muscles that occurred at least three seconds after the previous muscle movement. EMG activity had to be at least twice the amplitude of the background EMG. The bruxism episodes index (BEI) was measured by counting the number of bruxism episodes per hour of sleep. Phenotypes of bruxism were distinguished as follows: phasic, tonic and mixed [18]. The number of bruxism episodes per hour of sleep was used to categorize sleep bruxism and assigned as irrelevant (BEI < 2), mild to moderate (BEI 2–4) and severe (BEI > 4) [19].

### 2.3. Sample Collection and Determination of Blood and Urine Cadmium Concentration

Fasting venous blood samples from the ulnar vein and urine samples were obtained at 7:00 a.m., after twelve hours of overnight fasting. These blood samples were collected into polyethylene terephthalate (PET) plastic tubes (Becton, Dickinson and Company; Franklin Lakes, NJ, USA) with K_2_EDTA. Blood samples were stored at −70 °C until subsequent analyses were performed. Blood and urine samples were analyzed at the Main Laboratory of Wroclaw Medical University. Laboratory tests were performed according to the standard laboratory protocols of Wroclaw Medical University Teaching Hospital. The blood concentration of cadmium was determined by atomic absorption spectrophotometry with the use of a SOLAAR M6 (Thermo Elemental Ltd., London, UK) in an electrographite cuvette at λ = 228.8 nm, equipped with a Zeeman background correction system, in a certified atomic absorption laboratory at the University Hospital in Wroclaw, Poland. Values were measured in micrograms per deciliter (mg/dL).

For the analysis, the Stoeppler and Brandt method was used [20]. For trace metal analysis, the samples were deproteinized with 65% nitric acid (Suprapur; Merck KGaA, Darmstadt, Germany). Using ClinCal Whole Blood Calibrators (Cat.) and comparing the absorbance of the sample to a standard curve, the concentration of cadmium in the sample was determined. No. 9943) and control tests were performed (ClinChek Entire Blood Controls, level I, II, III; Cat. No. 8840–8843; Recipe Chemicals + Instruments GmbH, Munich, Germany). Within the framework of the German External Quality Assessment Scheme (G-EQUAS), external laboratory control was carried out with the assistance of the Intercomparison Program for Toxicological Analyses in Biological Materials (Institute and Out-Patient Clinic for Occupational, Social and Environmental Medicine of the Friedrich-Alexander University, Erlangen, Germany). The described method had a detection limit of 0.082 g/L.

### 2.4. Statistics

Statistical analysis was carried out based on the statistical software “Dell Statistica 13” (Dell Inc., Tulsa, OK, USA). The distribution of variables was examined with the Lilliefors and W-Shapiro–Wilk tests. In comparative analyses, subgroups were compared based on the median, first quartile or third quartile of the grouping variable. In the case of quantitative independent variables with a normal distribution, the t-test was used for further statistical analysis. In the case of variables with a distribution other than normal, the Mann–Whitney U test was used for quantitative independent variables. For qualitative independent variables, the maximum likelihood chi-square test was utilized. To determine the relationship between the studied variables, correlation and regression analysis was performed. In the case of quantitative variables with a normal distribution, Pearson’s r correlation coefficients were marked. For quantitative variables with a non-normal distribution, on the other hand, Spearman’s r coefficients were used. The parameters of the model obtained in the multivariable stepwise regression analysis were estimated using the least squares method. Results at the level of *p* < 0.05 were considered to be statistically significant.

## 3. Results

The data that were obtained from the study group and subsequently evaluated are demonstrated in Table 1. Overall, 44 patients were included in this study. The minimum sample group size required to conduct this study was estimated using a sample size calculator. The following estimation input conditions were used: population size: 3,000,000 (population size of the Lower Silesian Voivodeship); fraction size: 0.1 (assumed OSA prevalence in the Polish population); confidence level: 95% (default value); and maximum error: 10% (one of the typical values). The minimum required sample group size of 35 people was achieved.

The average age of the study group was 47.45 ± 13.69 years old. In total, 38.63% (*n* = 17) of the participants were female, while 61.36% (*n* = 27) were male; 86.36% (*n* = 38) of patients were current smokers; and 29 (67.44%) of the 44 the patients were diagnosed as sleep bruxers. Obstructive sleep apnea (OSA) was present in 70.45% (*n* = 31) patients in the study group. The degree of OSA was determined in each subject: 20.45% (*n* = 9) of cases were mild, 22.72% (*n* = 10) moderate and 27.27% (*n* = 12) of subjects had severe OSA. The mean blood and urine cadmium concentration in the study group was 0.37 ± 0.39 µg/L and 0.78 ± 0.63 µg/g of creatinine, respectively.

Patients were divided into two groups in accordance with their median cadmium blood concentration, namely, a low cadmium concentration group (<0.25) and a high cadmium concentration group (>0.25). In the low cadmium group, the average cadmium concentration was 0.14 ± 0.07, while in the high cadmium group it was 0.57 ± 0.46. The average SpO_2_% and minimal SpO_2_% were decreased in both of these groups. Statistical significance was present in SpO_2_ < 90% duration, minimal SpO_2_, average SpO_2_, REM sleep stage, N2 sleep stage, WASO, bruxism episode index and phasic bruxism (Table 2).

The results for cadmium concentration in the urine were compared as shown in Table 3. Correlation analysis was also performed and is shown in Table 4.

The result of the regression analysis is presented in Table 5. Age, high urine cadmium concentration, male gender and smoking are independent risk factors for increased AHI values (Table 5). Cadmium has not been found to be an independent risk factor for sleep bruxism.

## 4. Discussion

### 4.1. Evidence for Role of Cadmium in OSA

To the best of our knowledge, this is the first study investigating the role of cadmium on sleep parameters. In this study, cadmium was found to be a risk factor for an increased apnea/hypopnea index (AHI). As a result, our study shows that environmental exposure to cadmium may increase the risk of developing OSA.

We also determined that blood and urine cadmium concentration was correlated with blood oxygen saturation parameters. This suggests that cadmium exposure may affect the level of oxygen saturation in subjects with sleep disorders. As far as we know, no previous studies have ever investigated this specific relationship. However, it is known that cigarette smoking itself, which is the main source of cadmium exposure, has a negative influence on respiratory parameters [21].

Past studies evaluating the serum levels of heavy metals in patients with obstructive sleep apnea (OSA) revealed that, among others, the serum level of cadmium was increased in the study group (compared to controls), possibly due to oxidative stress and inflammation [22]. One such study, conducted by Asker et al., was based on recorded polygraphic measurements. No sleep architecture parameters were obtained in this study. However, despite this, the results are in agreement with the outcomes of the present study. Cadmium is known for its oxidative stress and inflammatory properties [7]. There is also a strong correlation between inflammation, oxidative stress and OSA, which has been proven in previous studies [23].

Cadmium may increase inflammation in the tissues of the respiratory tract, favoring the narrowing of the upper airway during sleep and affecting respiratory control stability/loop gain, the respiratory arousal threshold and upper airway muscle function via neurotoxic activity. On the other hand, hypoxia in OSA may affect the absorption of cadmium, thus the cadmium–OSA relationship may be bidirectional. However, these hypotheses require further research.

The influence of cigarette smoking on OSA development has recently been a subject of interest, with varying results in numerous studies [24]. Still, other proven risk factors for OSA include male gender, older age, obesity and craniofacial deformities [25]. No past studies have investigated the potential mechanisms (arising from cadmium exposure) that lead to the development of sleep apnea. To this day, the mechanisms underlying cadmium-induced airway pathologies have not yet been fully understood due to the complex relationship between biochemical and clinical outcomes. However, to the best of our knowledge, our study is the first to demonstrate that cadmium exposure is an independent OSA risk factor.

### 4.2. The Influence of Cadmium on Sleep Bruxism (SB) Intensity

No studies thus far have investigated the influence of cadmium exposure on SB intensity. In one of our previous studies, our team demonstrated that smoking, which is the main source of cadmium in humans, is a risk factor for SB development [26]. Our current study shows that cadmium itself is not a risk factor for the development of SB. However, we did find that the intensity of SB was increased in patients with a higher cadmium serum concentration, compared to subjects with a lower cadmium concentration. This result is not standalone though, and it is important to remember that many factors can influence the intensity of bruxism in patients with cadmium exposure, such as insomnia, psychological status or caffeine consumption. Sleep bruxism and obstructive sleep apnea are also two strongly connected disorders that can even co-occur [27]. Besides its nicotine content, various toxic components are present in a single cigarette, each of which may affect the intensity of bruxism. A host of reactive oxygen and nitrogen species (ROS and RNS) may alter the intensity of bruxism and endothelial function. The endothelium regulates the inflammatory state in a human’s body and cigarette smoking is known to lead to endothelial dysfunction [28]. Thus, many proinflammatory and toxic compounds can affect the development of sleep bruxism but, as explained above, not cadmium.

In general, cigarettes act on the sympathetic nervous system by activating it [29]. On the contrary, though, in sleep bruxism the trigemino-cardiac reflex (TCR) plays an important role in the regulation of the autonomic nervous system. The activation of the TCR results in the suppression of sympathetic nervous activity, while parasympathetic activity is activated as a sort of defense mechanism [30]. As a result, the intensity of sleep bruxism is increased in smokers, however not due to the cadmium content, but rather due to other factors.

### 4.3. Evidence for the Role of Cadmium in Sleep Architecture Alterations

No research thus far has investigated the influence of cadmium on sleep architecture in humans. Only one study has explored the relationship between sleep and cadmium exposure in rats. However, this study only made use of an EEG investigation, which revealed that a high concentration of cadmium in drinking water led to an increase in non-REM sleep and a decrease in rhythms of locomotor activity [31]. Animal-based studies found that cadmium can influence sleep architecture and sleep duration, while acute cadmium exposure seemed to have an impact on the circadian rhythm [32,33,34,35]. Our results are consistent with previous investigations.

Past studies investigating the neurotoxic consequences of cadmium determined that cadmium exposure induces memory impairment and results in a decreased attention span in humans [36]. Interestingly, our present study demonstrates that cadmium exposure impacts the REM sleep stage by decreasing the duration of this phase of sleep. The REM stage of sleep participates in memory consolidation [37] and emotional processing [38]. These mechanisms can potentially be affected in patients exposed to cadmium. Further investigations are needed to explain this correlation.

We also found a positive correlation between cadmium content in blood/urine and the frequency of arousals during sleep. Higher concentrations of cadmium in the blood and urine were found among subjects with an increased arousal index. It is known that arousals favor sleep fragmentation and sleep architecture alterations [39]. It is worth noting that fragmented sleep is a known cardiovascular risk factor [40].

Our study group had environmental exposure to cadmium. The mean blood cadmium concentration was 0.37 ± 0.39 µg/g, while the concentration in the urine was 0.78 ± 0.63 µg/g of creatinine. In the general population, geometric cadmium levels in blood have been estimated to be around 0.315 ng/L, while in urine these levels are approximately 0.193 ng/g of creatinine [41]. In our study, these biomarkers were higher, and this result confirms environmental cadmium exposure in our patients.

In conclusion, the complexity of cadmium-dependent sleep architecture outcomes remains to be clarified and further studies on the various mechanisms that result in sleep pattern changes are needed. It may help to develop new methods of OSA treatments. However, in conclusion of our study, patients with OSA should avoid environmental and occupational cadmium exposure.

### 4.4. Study Strengths

Full-night audio–video polysomnography, which is the gold standard in sleep examination, was performed in the whole study group. In addition, both blood and urine samples were obtained and measured to determine cadmium concentration. Blood and urine are known to be exposure biomarkers [3]. However, blood cadmium is more likely to indicate recent cadmium exposure, while cadmium found in the urine has been linked to chronic or past exposure and may indicate the total cadmium body burden [42].

### 4.5. Study Limitations

The relatively small study group (*n* = 44) was the major limitation of this study. In addition, there was no adaptive night prior to conducting the PSG examination due to a limited capacity in the sleep laboratory, and due to restrictions associated with the organization of the hospital. This study was not intended to show an influence of cigarette smoking on sleep parameters, but to investigate blood and urine cadmium influence on sleep architecture. However, smoking may explain some changes in sleep architecture and further research is needed to determine this relation.

## 5. Conclusions

1. Cadmium is an independent factor for increased AHI, similarly to age, gender and smoking status.
2. Cadmium is not a risk factor for sleep bruxism.
3. Cadmium favors sleep disturbances, including sleep fragmentation, and results in the limitation of the duration of the REM sleep stage.

## Figures and Tables

**Table 1:** Polysomnographic parameters in the study group.

Parameter	Average	Median	Minimum	Maximum
AHI (*n*/h)	24.22 ± 25.34	14.85	0.50	86.20
ODI (*n*/h)	23.45 ± 24.11	13.65	0.00	86.70
Snore (% of TST)	25.79 ± 22.29	26.75	0.00	75.40
Average SpO_2_ (%)	91.57 ± 4.50	92.55	74.60	95.80
Minimal SpO_2_ (%)	79.82 ± 10.46	83.00	51.00	93.00
SpO_2_ duration <90% (%)	16.01 ± 25.02	4.10	0.00	86.60
SL (min)	19.97 ± 15.79	13.65	1.00	64.60
WASO (min)	59.03 ± 44.11	44.50	7.50	186.10
SE (%)	82.80 ± 12.24	85.25	36.50	97.40
N1 (% of TST)	7.14 ± 7.74	3.40	0.30	32.70
N2 (% of TST)	52.03 ± 21.45	50.30	28.60	181.00
N3 (% of TST)	25.37 ± 20.55	23.00	6.40	146.50
REM (% of TST)	21.31 ± 6.53	21.70	0.00	34.40
ArI (*n*/h)	7.96 ± 13.57	3.75	0.10	88.30
BEI (*n*/h)	4.17 ± 3.29	3.30	0.00	13.60
Phasic bruxism episode index (*n*/h)	1.79 ± 2.18	1.40	0.00	10.70
Tonic bruxism episode index (*n*/h)	1.60 ± 1.39	1.10	0.00	5.50
Mixed bruxism episode index (*n*/h)	0.81 ± 0.80	0.60	0.00	3.40
AI (*n*/h)	11.96 ± 17.80	3.05	0.00	69.20
OA (*n*/h)	9.89 ± 15.60	2.00	0.00	62.40
MA (*n*/h)	0.88 ± 2.39	0.00	0.00	14.20
CA (*n*/h)	1.19 ± 2.77	0.35	0.00	16.80
HI (*n*/h)	12.26 ± 12.15	8.35	0.10	50.60
Average pulse (bpm)	62.47 ± 8.83	61.35	48.70	94.40
Maximal pulse (bpm)	94.95 ± 14.28	96.50	64.00	140.00
Minimal pulse (bpm)	46.29 ± 11.37	48.00	4.60	71.0

AHI: apnea–hypopnea index; ODI: oxygen desaturation index; SL: sleep latency; WASO: wake after sleep onset; SE: sleep efficiency; REM: rapid eye movement; TST: total sleep time; ArI: arousal index; BEI: bruxism episode index; AI: apnea index; OA: obstructive apneas; MA: mixed apneas; CA: central apneas; HI: hypopneas index.

**Table 2:** Blood cadmium concentration (micrograms/g) regarding median (Me), lower (Q1) and upper quartile (Q3).

Paramerer	Average ≥ Me	Average < Me	*p*	Average ≥ Q1	Average < Q1	*p*	Average <Q3	Average ≥Q3	*p*
Cadmium blood concentration (µg/L)	**0.57 ± 0.46**	**0.14 ± 0.07**	**0.000**	**0.46 ± 0.42**	**0.09 ± 0.03**	**0.006**	**0.20 ± 0.10**	**0.85 ± 0.54**	**0.000**
AHI (*n*/h)	30.49 ± 26.72	17.35 ± 22.38	0.086	27.92 ± 26.70	13.14 ± 17.35	0.094	20.13 ± 21.99	36.50 ± 31.52	0.063
ODI (*n*/h)	29.34 ± 25.69	17.00 ± 20.97	0.090	27.24 ± 25.59	12.09 ± 14.63	0.071	19.74 ± 20.49	34.59 ± 31.19	0.076
Snore (%)	27.00 ± 24.58	24.46 ± 20.01	0.710	27.48 ± 22.89	20.73 ± 20.56	0.391	22.15 ± 19.04	36.72 ± 28.31	0.060
Average SpO_2_ (%)	**90.29 ± 5.58**	**92.97 ± 2.33**	**0.048**	90.88 ± 4.94	93.62 ± 1.72	0.081	**92.58 ± 2.81**	**88.52 ± 6.94**	**0.008**
Minimal SpO_2_ (%)	**76.04 ± 12.13**	**83.95 ± 6.24**	**0.010**	**77.76 ± 11.03**	**86.00 ± 4.96**	**0.022**	**82.94 ± 6.62**	**70.45 ± 14.19**	**0.000**
SpO_2_ duration <90% (min)	20.03 ± 29.24	11.60 ± 19.15	0.270	19.30 ± 27.48	6.12 ± 11.47	0.132	**10.22 ± 16.15**	**33.38 ± 37.61**	**0.006**
SL (min)	21.76 ± 18.74	18.01 ± 11.91	0.438	20.34 ± 16.84	18.85 ± 12.74	0.789	19.18 ± 13.12	22.33 ± 22.64	0.573
WASO (min)	64.73 ± 41.07	52.79 ± 47.42	0.376	**67.04 ± 46.18**	**35.01 ± 26.38**	**0.035**	56.71 ± 43.45	65.99 ± 47.47	0.552
SE (%)	82.17 ± 9.90	83.49 ± 14.61	0.726	82.29 ± 10.57	84.32 ± 16.84	0.640	83.21 ± 12.77	81.55 ± 10.96	0.702
N1 (% of TST)	6.77 ± 6.79	7.55 ± 8.82	0.743	7.06 ± 7.83	7.38 ± 7.82	0.907	7.45 ± 8.45	6.20 ± 5.29	0.647
N2 (% of TST)	49.49 ± 9.09	54.80 ± 29.72	0.419	**48.07 ± 8.84**	**63.88 ± 39.02**	**0.033**	51.91 ± 24.38	52.37 ± 8.75	0.951
N3 (% of TST)	23.30 ± 10.39	27.62 ± 27.91	0.493	23.30 ± 9.30	31.55 ± 38.51	0.253	25.97 ± 23.10	23.55 ± 10.16	0.740
REM (% of TST)	20.41 ± 7.16	22.29 ± 5.77	0.348	21.54 ± 7.04	20.61 ± 4.87	0.687	**22.46 ± 5.25**	**17.85 ± 8.79**	**0.041**
Bruxism episodes index (*n*/h)	4.20 ± 3.25	4.14 ± 3.41	0.955	4.64 ± 3.23	2.80 ± 3.20	0.111	**3.60 ± 2.93**	**6.03 ± 3.87**	**0.039**
Phasic bruxism	2.06 ± 2.38	1.50 ± 1.96	0.407	2.02 ± 2.23	1.14 ± 1.96	0.253	**1.34 ± 1.65**	**3.27 ± 3.05**	**0.012**
Tonic bruxism	1.48 ± 1.04	1.73 ± 1.69	0.559	1.74 ± 1.28	1.19 ± 1.66	0.261	1.55 ± 1.47	1.75 ± 1.13	0.701
Mixed bruxism	0.70 ± 0.63	0.94 ± 0.94	0.323	0.93 ± 0.84	0.49 ± 0.56	0.119	0.74 ± 0.83	1.05 ± 0.65	0.289
ArI (*n*/h)	9.53 ± 18.08	6.23 ± 5.53	0.427	8.30 ± 15.43	6.93 ± 5.32	0.775	6.38 ± 5.83	12.70 ± 25.50	0.184
AI (*n*/h)	14.81 ± 19.43	8.84 ± 15.70	0.272	13.77 ± 19.13	6.55 ± 12.19	0.249	9.85 ± 15.61	18.30 ± 22.91	0.176
OA (*n*/h)	11.72 ± 16.39	7.89 ± 14.82	0.422	11.26 ± 16.57	5.78 ± 11.97	0.319	7.62 ± 12.78	16.71 ± 21.34	0.094
MA (*n*/h)	1.22 ± 3.14	0.50 ± 1.09	0.330	1.02 ± 2.68	0.46 ± 1.22	0.515	0.84 ± 2.56	0.99 ± 1.92	0.858
CA (*n*/h)	1.88 ± 3.71	0.43 ± 0.51	0.083	1.49 ± 3.15	0.28 ± 0.24	0.216	1.38 ± 3.16	0.62 ± 0.78	0.439
HI (*n*/h)	**15.68 ± 14.26**	**8.51 ± 8.09**	**0.049**	14.15 ± 12.97	6.58 ± 7.01	0.073	10.28 ± 10.32	18.21 ± 15.56	0.060

Me: median; Q: quartile; AHI: apnea–hypopnea index; ODI: oxygen desaturation index; SL: sleep latency; WASO: wake after sleep onset; SE: sleep efficiency; REM: rapid eye movement; TST: total sleep time; ArI: arousal index; BEI: bruxism episode index; AI: apnea index; OA: obstructive apneas; MA: mixed apneas; CA: central apneas; HI: hypopneas index; statistically significant values are shown in bold (*p* < 0.05).

**Table 3:** Urine cadmium concentration (micrograms/g creatinine) regarding median, lower (Q1) and upper quartile (Q3).

Parameter	Average < Me	Average ≥ Me	*p*	Average < Q1	Average ≥ Q1	*p*	Average < Q3	Average ≥ Q3	*p*
Cadmium urine concentration (µg/g of creatinine)	**0.34 ± 0.11**	**1.22 ± 0.62**	**0.000**	**0.24 ± 0.05**	**0.94 ± 0.63**	**0.001**	**0.47 ± 0.23**	**1.70 ± 0.54**	**0.000**
AHI (*n*/h)	16.85 ± 20.31	31.59 ± 28.09	0.053	13.94 ± 18.00	27.24 ± 26.59	0.147	20.02 ± 21.25	36.81 ± 32.92	0.056
ODI (*n*/h)	**15.76 ± 18.82**	**31.14 ± 26.68**	**0.033**	13.13 ± 15.00	26.49 ± 25.57	0.125	19.49 ± 19.77	35.34 ± 32.26	0.058
Snore (%)	25.50 ± 21.83	26.08 ± 23.25	0.933	16.74 ± 22.37	28.45 ± 21.89	0.146	26.30 ± 21.26	24.25 ± 26.20	0.794
Average SpO_2_ (%)	**93.13 ± 2.20**	**90.00 ± 5.61**	**0.019**	93.57 ± 1.88	90.98 ± 4.88	0.110	**92.58 ± 2.75**	**88.53 ± 7.02**	**0.008**
Minimal SpO_2_ (%)	**83.64 ± 7.13**	**76.00 ± 11.94**	**0.014**	84.80 ± 5.35	78.35 ± 11.18	0.087	**82.42 ± 7.15**	**72.00 ± 14.72**	**0.003**
SpO_2_ duration <90% (% of TST)	9.09 ± 17.30	22.93 ± 29.70	0.066	9.26 ± 11.33	17.99 ± 27.62	0.338	**9.70 ± 15.55**	**34.94 ± 37.32**	**0.003**
SL (min)	18.64 ± 14.18	21.30 ± 17.48	0.582	26.03 ± 16.90	18.19 ± 15.24	0.170	19.42 ± 14.16	21.62 ± 20.63	0.694
WASO (min)	51.02 ± 41.30	67.05 ± 46.30	0.232	52.01 ± 41.65	61.10 ± 45.20	0.573	54.00 ± 42.31	74.14 ± 48.00	0.193
SE (%)	84.00 ± 14.26	81.59 ± 10.01	0.519	79.21 ± 18.12	83.85 ± 10.03	0.297	83.76 ± 12.99	79.91 ± 9.58	0.372
N1 (% of TST)	8.13 ± 9.03	6.15 ± 6.25	0.402	7.73 ± 7.61	6.97 ± 7.88	0.788	7.10 ± 8.06	7.27 ± 7.04	0.949
N2 (% of TST)	54.99 ± 28.66	49.06 ± 10.12	0.366	62.24 ± 42.09	49.02 ± 8.69	0.087	52.56 ± 23.83	50.42 ± 12.58	0.778
N3 (% of TST)	26.85 ± 27.73	23.88 ± 9.54	0.637	33.69 ± 40.26	22.92 ± 9.00	0.147	26.11 ± 22.80	23.15 ± 12.06	0.684
REM (% of TST)	21.74 ± 5.09	20.87 ± 7.80	0.664	22.09 ± 6.54	21.08 ± 6.60	0.671	22.03 ± 4.80	19.15 ± 10.10	0.208
Bruxism episode index (*n*/h)	4.11 ± 3.92	4.22 ± 2.56	0.914	3.65 ± 3.41	4.32 ± 3.29	0.576	3.98 ± 3.41	4.79 ± 2.92	0.501
Phasic bruxism episode index (*n*/h)	1.81 ± 2.74	1.77 ± 1.43	0.945	1.44 ± 1.99	1.90 ± 22.25	0.567	1.62 ± 2.32	2.35 ± 1.61	0.360
Tonic bruxism episode index (*n*/h)	1.55 ± 1.62	1.65 ± 1.12	0.812	1.55 ± 1.87	1.62 ± 1.24	0.898	1.61 ± 1.47	1.58 ± 1.12	0.959
Mixed bruxism episode index (*n*/h)	0.77 ± 0.83	0.86 ± 0.77	0.704	0.68 ± 0.66	0.85 ± 0.84	0.549	0.78 ± 0.84	0.94 ± 0.66	0.573
ArI (*n*/h)	6.99 ± 5.50	8.93 ± 18.57	0.640	6.51 ± 5.68	8.39 ± 15.18	0.706	6.29 ± 5.01	12.97 ± 25.98	0.160
AI(*n*/h)	8.26 ± 14.03	15.66 ± 20.58	0.171	7.09 ± 12.71	13.39 ± 18.97	0.331	9.95 ± 15.73	17.99 ± 22.75	0.198
OAI (*n*/h)	7.19 ± 13.21	12.60 ± 17.57	0.255	6.07 ± 12.59	11.01 ± 16.38	0.385	7.94 ± 12.91	15.75 ± 21.52	0.153
MAI (*n*/h)	0.52 ± 1.08	1.23 ± 3.21	0.332	0.53 ± 1.26	0.98 ± 2.64	0.608	0.89 ± 2.58	0.85 ± 1.82	0.960
CAI (*n*/h)	0.55 ± 0.60	1.83 ± 3.81	0.127	0.50 ± 0.47	1.39 ± 3.13	0.380	1.12 ± 2.94	1.39 ± 2.31	0.781
HI (*n*/h)	**8.60 ± 7.95**	**15.91 ± 14.53**	**0.045**	6.86 ± 7.28	13.85 ± 12.90	0.111	**10.08 ± 8.40**	**18.81 ± 18.60**	**0.037**
Average pulse (bpm)	**59.56 ± 9.30**	**65.37 ± 7.45**	**0.027**	59.58 ± 13.22	63.31 ± 7.12	0.244	61.00 ± 9.15	66.86 ± 6.28	0.055
Maximal pulse (bpm)	94.64 ± 11.09	47.95 ± 11.64	0.085	91.70 ± 20.09	95.91 ± 12.30	0.419	94.88 ± 15.51	45.36 ± 12.06	0.952
Minimal pulse (bpm)	44.62 ± 11.09	47.95 ± 11.64	0.336	41.16 ± 13.23	47.79 ± 10.50	0.105	46.59 ± 11.30	45.36 ± 12.06	0.760

Me: median; Q: quartile; AHI: apnea–hypopnea index; ODI: oxygen desaturation index; SL: sleep latency; WASO: wake after sleep onset; SE: sleep efficiency; REM: rapid eye movement; TST: total sleep time; ArI: arousal index; BEI: bruxism episode index; AI: apnea index; OAI: obstructive apneas index; MAI: mixed apneas index; CAI: central apneas index; HI: hypopneas index; statistically significant values are shown in bold (*p* < 0.05).

**Table 4:** Correlation analysis.

Variable	Cadmium Blood Concentration (µg/L)	*p*	Cadmium Urine Concentration [µg/g of Creatinine]	*p*
AHI (*n*/h)	**0.44**	**<0.05**	**0.36**	**<0.05**
ODI (*n*/h)	**0.44**	**<0.05**	**0.38**	**<0.05**
Snore (%)	**0.33**	**<0.05**	−0.02	>0.05
Average SpO_2_ (%)	**−0.57**	**<0.05**	**−0.56**	**<0.05**
SpO_2_ duration <90% (% of TST)	**0.47**	**<0.05**	**0.50**	**<0.05**
Average desaturation	**0.45**	**<0.05**	**0.52**	**<0.05**
Minimal SpO_2_ (%)	**−0.54**	**<0.05**	**−0.53**	**<0.05**
Minimal pulse	**−0.46**	**<0.05**	0.07	>0.05
REM (% of TST)	**−0.44**	**<0.05**	−0.08	>0.05
ArI (*n*/h)	**0.60**	**<0.05**	**0.42**	**<0.05**
AI (*n*/h)	0.29	>0.05	**0.32**	**<0.05**
OAI (*n*/h)	**0.33**	**<0.05**	**0.35**	**<0.05**
HI (*n*/h)	**0.48**	**<0.05**	0.29	>0.05

AHI: apnea–hypopnea index; ODI: oxygen desaturation index; REM: rapid eye movement; ArI: arousal index; AI: apnea index; OAI: obstructive apneas index; HI: hypopneas index; statistically significant values are shown in bold (*p* < 0.05).

**Table 5:** The regression model of obstructive sleep apnea predictors in the study population.

Parameter	RC	SEM of RC	*p*
Intercept	**−37.024**	**15.156**	**0.0193**
Age	**0.480**	**0.234**	**0.0438**
Cadmium urine concentration (µg/g of creatinine)	**15.795**	**5.921**	**0. 0111**
Male	**22.955**	**7.106**	**0.0025**
Smoking	**11.765**	**3.226**	**0.0257**

RC—regression coefficient; SEM of RC—standard error mean of RC; statistically significant values are shown in bold (*p* < 0.05).

## Data Availability

The data supporting the findings of this study are available on request from the corresponding author and are not publicly available due to privacy or ethical restrictions.
